# Breast self-examination and associated factors among women in Wolaita Sodo, Ethiopia: a community-based cross-sectional study

**DOI:** 10.1186/s12905-020-01042-1

**Published:** 2020-08-08

**Authors:** Temesgen Lera, Aman Beyene, Befekadu Bekele, Solomon Abreha

**Affiliations:** 1 School of Public Health, Wolaita Sodo University, Wolaita, Ethiopia; 2 South Ethiopia Nations Development Association, Wolaita Sodo, Wolaita, Ethiopia

**Keywords:** Breast self-examination (BSE), Breast cancer, Wolaita Sodo, Ethiopia

## Abstract

**Background:**

The early detection of breast cancer plays an important role in decreasing morbidity and mortality of breast cancer. Breast self-examination (BSE) is one screening method used for the early detection of breast cancer. BSE involves the woman looking at and feeling each breast for possible lumps, distortions, or swellings. BSE is a simple exercise that can potentially save women’s lives, but BSE receives relatively little attention and no study has yet addressed BSE at the community level. Here we assessed BSE and associated factors among women aged 20–65 years in Wolaita Sodo city, Ethiopia.

**Methods:**

This was a community-based, cross-sectional study. Systematic random sampling was used to select 626 women aged 20–65 years old. Data were collected using a pre-tested and structured questionnaire. Data were recorded using EpiData version 3.5.1 and exported to SPSS version 21 for cleaning and statistical analysis. Bivariable analysis was performed, and variables with a *p*-value < 0.25 were used in multiple logistic regression analysis. Multiple logistic regression was employed, and variables with *p*-values < 0.05 were considered statically significant.

**Results:**

A total of 629 women aged between 20 and 65 years were included in the study. Over half (60.9%) of participants were aged between 20 and 29 years, and 8.2% were < 50 years old. Women who mentioned BSE as a method for the early detection of breast problems were 6.36-times (95% CI: 3.72, 10.71) more likely to perform BSE than those who reported that they did not know of any method. Those who had breast fed for 13–24 months were 2.43 times (95% CI: 1.28, 4.59) more likely to examine their breasts than those who breast fed for different durations or used other methods. Employed study participants were 3.13-times (95% CI: 1.14, 8.58) more likely to practice BSE than those who were not employed. Likewise, students were 3.73-times (95% CI: 1.19, 11.73) more likely to perform BSE.

**Conclusions:**

In our sample, women’s practice of BSE was relatively low. Knowledge of BSE, breastfeeding up to 24 months, being employed, and being a student were factors affecting performing BSE. Educating girls and increasing awareness, including through electronic media, are important to encourage BSE and improve breast cancer outcomes.

## Plain English summary

Detecting breast cancer early is important for decreasing its associated morbidity and mortality. Breast self-examination (BSE) is a screening method used to detect breast cancer early. In this study, respondents were asked via a closed-ended structured questionnaire conducted through face-to-face interviews whether or not they practiced breast self-examination regularly. Six hundred twenty-nine women participated, with a response rate of 100%. 76% of study participants were married, and 49.8% percent of women were housewives. We found that the prevalence of BSE among this sample of women was 34.5%. Knowledge of BSE, breastfeeding up to 24 months, being employed, and being a student were factors associated with performing BSE. Therefore, educating girls and increasing awareness, including through electronic media, are important to encourage BSE and improve breast cancer outcomes.

## Background

Breast cancer is an issue of public health importance in both developed and developing nations. Due to its high prevalence, breast cancer places significant pressure on health system resources and incurs significant healthcare costs. Breast cancer is the second leading cause of death among women globally, more than a million new cases detected yearly, accounting for 10.9% of all cancer cases, second to lung cancer [1, 2]. Its occurrence is growing both in developed and developing regions. An estimated 636,000 new cases were identified in high-resource countries, while 514,000 cases were identified in low- and middle-resource countries in 2008. Breast cancer is the most recurrent cause of death among women both in developing and developed counties, with an estimated 269,000 and 189,000 losses, respectively. Seventy percent of all breast cancer cases will be in low- and middle-resource countries by 2020 [3] globally. The occurrence of breast cancer varies across the African region, ranging from 19.3 per 100,000 per year in Eastern Africa to 38.1 per 100,000 per year in Southern Africa [4].

Breast self-examination (BSE) is a breast cancer screening method that involves the woman looking at and feeling her own breasts for possible lumps, distortions, or swellings. BSE is a simple exercise that can potentially save women’s lives. BSE is recommended for every woman from the age of 20 years onwards, and BSE is recommended to be performed for 20 min every month [5]. However, women in developing countries are known not to perform BSE for various reasons [1]. A woman who performs BSE may be more motivated to seek medical attention, including clinical breast examination (CBE) and mammography [6].

In Ethiopia, over half of women with breast cancer are aged 50 and younger. It has been shown that 69.6% of patients ignore their initial symptoms for an average of over 1.5 years [7]. BSE is still recommended as a general approach to increasing breast health awareness and detecting any anomalies because it is free, painless, and easy to practice [5]. Furthermore, the American Cancer Society also recommends that women, from age 20, should be educated on the advantages and disadvantages of performing monthly BSE [8].

Breast cancer in low- to middle-income countries tends to present late and has poor clinical outcomes due to several factors such as unequal access to prompt high-quality treatment and a lack of early screening [3]. Despite the fact that breast cancer has recently become a leading cause of mortality in young women, especially those in urban areas, the Ethiopian health system has traditionally concentrated on communicable disease prevention as a public health priority [7]. Furthermore, there are very few reports measuring BSE at the population level. Here we addressed this knowledge gap by assessing BSE and its associated factors in women aged 20–65 years in Wolaita Sodo city, Ethiopia, to identify the need for information on BSE in Ethiopia.

## Methods

### Study setting and design

The study was carried out in Wolaita Sodo city. The city has a total population of 250,521 (male 79,871 (52%), female 73,650 (48%)), and the city has three sub-cities, 18 kebeles, three health centers, one Ministry of Health-owned hospital, and one private hospital. The city is located 160 km from the regional city Hawassa and 327 km from Addis Ababa, the capital of Ethiopia [9]. A community-based cross-sectional study design was employed.

#### Source population

All women aged 20-65 years were considered as a source population.

#### Study population

Houses in selected kebeles were included by systematic random sampling, and study unit was selected by the simple random sampling technique.

#### Inclusion and exclusion criteria

Women age 20–60 years were included in the study, and women who were seriously ill during the period of data collection, had known breast cancer, and those not willing to participate in the study were excluded.

#### Sample size and sampling procedure

Sample size was calculated with EPidata statistical software version 3.03 using the single population proportion statistical formula:

$$ \mathrm{N}=\mathrm{Z}{(1.96)}^2\ \mathrm{P}\left(1-\mathrm{P}\right)/{\mathrm{d}}^2 $$

with the assumptions z = 1.96 at a 95% confidence level; the P-prevalence of BSE was taken as 53.6% (0.536) from a previous study [10]; and the non-response rate was 10%, confidence levels of 95%, and a 5% margin of error.

Therefore, the calculated sample size was 572 and, after considering a 10% non-response rate, the final sample size was 629.

### Sampling procedure

Multi-stage sampling was used to select study respondents. First, among the 18 kebeles in the city, 6 kebeles were randomly selected by simple random sampling to represent all kebeles. The source population in each selected kebele was identified from Wolaita Sodo Finance Economic Development Department data [9]. The sample size allocated to the selected kebeles was proportional to the source population in the kebele. The sampling interval was calculated by diving the source population by our sample [(N/n) = 15,098/629 = 24]. The first household was selected by simple random sampling from 1 to 24 listed households, and the 10th household was chosen randomly.

**Fig. 1:**
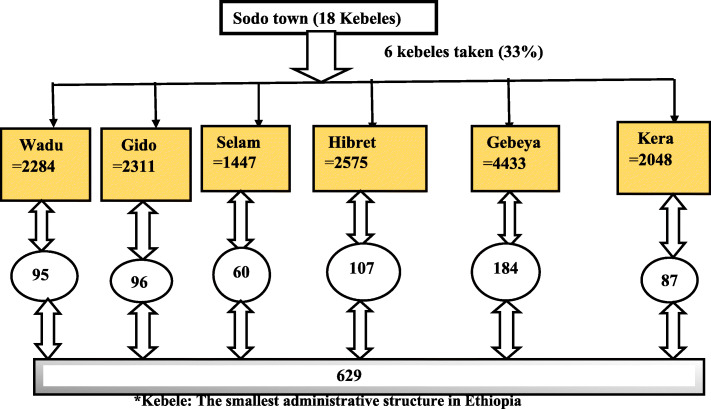
Sampling procedure of the study

Sampling frame (household)-containing lists of the population in the selected kebeles were obtained, and every 24th house was visited to select the study population by systematic random sampling until reaching the intended sample size for a given kebele. The respondents from each selected household were taken by simple random sampling whenever there was more than one eligible woman in a selected household (Fig. 1).

### Data collection procedure

Structured, pre-tested, and interviewer-administered questionnaires were used. The questions included sociodemographic characteristics and BSE-related issues. The questionnaires were adapted from the Ethiopian Development and Health Survey (EDHS) and the published literature. Data were collected between 24 November 2018 and 2 December 2018 by trained data collectors. Data were collected through face-to-face interviews, maintaining the pre-determined sampling intervals. The data collectors informed the respondents about all the details of the research and procedures, what was expected of them, and the potential risks and benefits in order to encourage accurate and honest responses. When the woman was not available at the first visit, data collectors arranged alternative visits. If a woman was still not available on second visits or declined to participate, the household was skipped and the immediate next household in the sampling frame was considered.

### Data quality management

Before data collection, the questionnaire was first prepared in English and translated into Amharic and back to English to maintain consistency. Data collectors and supervisors received 2 days of training by the principal investigator before data collection.

A pre-test was conducted in Dilbetigil kebele, which was not one of the selected kebeles, and 5% of the total sample size was tested. Based on the pre-test, questionnaires were revised, edited, and necessary corrections made. Daily checks of data for completeness and consistency were performed during data collection.

### Data analysis procedures

Data were entered into EpiData version 3.1 and cleaned to check for accuracy, consistency, completeness, and values, and any identified error was corrected.

Data were exported into SPSS version 21 (IBM Statistics, Chicago, IL) for analysis. Descriptive statistics were performed. Bivariable analyses were computed, and variables with *p*-values < 0.25 were considered candidates for multiple logistic regression analysis. Multiple logistic regression analysis was performed, and variables with p-values ≤0.05 were considered statistically significant. Adjusted odds ratios (AOR) with 95% confidence intervals (CI) were calculated.

### Ethical issues

Ethical clearance was obtained from Wolaita Sodo University Institutional Review Board (IRB). Written permission was obtained from Sodo City Health Department. During data collection, all respondents were asked for permission, and informed consent was obtained from each study participant.

### Operational definitions

#### Breast self-examination (BSE)

The self-examination of the breasts to identify any changes in the breasts [11].

#### Breast self-examination performed

If the woman performed BSE at least once in the last 12 months.

#### Age 20 to 65

American Cancer Society-recommended BSE for women aged 20 or older, and mammography for women aged 40 or older [8].

## Results

### Sociodemographic characteristics of the subjects

Six hundred and twenty-nine women were interviewed and subjected to analysis. The participants were aged between 20 and 65 years. 60.9% of participants were aged 20–29 years, and 8.2% were aged over 50 years. Eighty-seven percent of participants were Wolaita in ethnicity, and 70.6% were protestant. Three hundred and thirty-eight respondents had completed secondary education, and 478 study participants were married (Table 1).

**Table 1:** Sociodemographic characteristics of the women in Wolaita Sodo, 2019 (*n* = 629)

Variables/characteristics	Frequency (%)
Age distribution of the women	
20–29 years	383 (60.9)
30–39 years	139 (22.1)
v40–49 years	55 (8.7)
v ≥ 50 years	52 (8.3)
Marital status	
Never married	113 (18)
Married	478 (76)
Divorce	17 (2.7)
Widowed	21 (3.3)
Participant’s education	
No education	73 (11.6)
Primary	218 (34.8)
Secondary	179 (28.4)
Higher education	159 (25.3)
Husband’s education	
No education	20 (4.2)
Primary	131 (27.4)
Secondary	148 (31)
Higher education	179 (37.4)
Religion	
Protestant	444 (70.6)
Orthodox	131 (20.8)
Muslim	24 (3.8)
Catholic	16 (2.5)
Others	14 (2.2)
Ethnicity	
Wolaita	549 (87.3)
Gamo	32 (5.1)
Garage	18 (2.9)
Amhara	16 (2.5)
vOthers	14 (2.2)
Occupational status of the women	
House wife	312 (49.6)
Employee	133 (21.1)
Merchant	74 (11.8)
Student	54 (8.6)
Other	56 (8.9)
Age at which first pregnancy occurred	
15–24 years	397 (76.9)
25–34 years	111 (21.5)
35–44 years	2 (0.4)
≥ 45 years	6 (1.2)
Duration of breastfeeding	
Birth-12 months	77 (15.4)
13–24 months	280 (56.6)
25–34 months	141 (28)
Number of children	
None	23 (4.7)
One	104 (21.4)
Two	115 (23.7)
Three or more	244 (50.2)

### Knowledge and practice of BSE and information sources

**Fig. 2:**
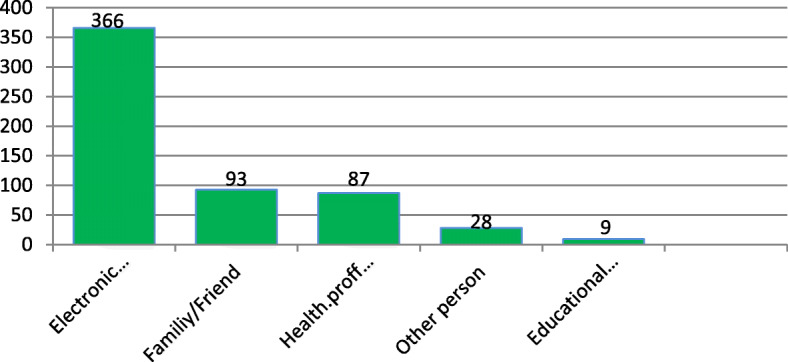
Breast cancer information sources among women in Sodo city, 2019

Among the respondents, 94% knew (heard or read) about breast cancer and their main source of information was electronic media (62.4%). The contribution of health professionals as a source of breast cancer information was found to be 14.7%. Electronic media, family/friends, and health workers were reported as major sources of information. Forty five respondents reported receiving information on breast cancer from other sources like journals, books, and non-governmental organizations (Fig. 2).

The reported methods of breast cancer screening were clinical breast examination (45.3%) and BSE (18%) and 36.5% women responded that they did not know of any method of breast cancer screening. Of those who had heard about breast cancer, 46% had also heard about BSE and, among study participants who had received information on BSE, 79.8% had performed BSE and 71.6% reported they regularly performed it. Among those who had ever heard of breast cancer, 92% knew (heard) their family history of breast cancer (Table 2).

**Table 2:** Knowledge and practice of BSE and main information sources among women in Wolaita Sodo, 2019 (*n* = 629)

Characteristics/variables	Frequency (%)
Ever heard of breast cancer	
Yes	591 (94)
No	38 (6)
Source of information	
Electronic media	366 (62)
Journal/brochure/leaflet/magazine	4 (0.8)
Books	3 (0.5)
Educational institution	9 (1.4)
Non-governmental organizations	1 (0.2)
Health workers	87 (14.7)
Family/friend	93 (15.8)
Other person	28 (4.7)
Previously heard of BSE	
Yes	272 (46)
No	319 (54)
Early detection method for breast cancer	
Breast self-examination (BSE)	107 (18)
Clinical breast examination (CBE)	268 (45.3)
I don’t know	216 (36.5)
Performed breast self-examination	
Yes	217 (78)
No	55 (20.2)
Still perform breast self-examination	
Yes	195 (90)
No	22 (10)
Knowledge that early detection of breast cancer improves chances of survival	
Yes	570 (96.7)
No	13 (2)
I don’t know	8 (1.3)
Family history of breast cancer	
Yes	14 (2.4)
No	523 (88.6)
I don’t know	54 (9)
Personal history of having benign breast lamp	
Yes	20 (3.3)
No	197 (33.3)
I don’t know	374 (63.4)
Knowledge of someone suffering from breast cancer	
Yes	116 (20)
No	475 (80)
Ever nursed a breast cancer patient	
Yes	4 (1)
No	587 (99)
Had close contact with a person with a benign breast lamp	
Yes	18 (3)
No	573 (97)
Knowledge of personal status of other body part cancer	
Yes	446 (75)
No	145 (25)
Position of BSE	
Standing	49 (22.4)
Lying	45 (21)
Sitting	16 (7.3)
Standing and lying	107 (49.3)
Technical knowledge of BSE	
With palm and three middle fingers	35 (16)
Simply touch and feel	157 (72.5)
I don’t know	25 (11.5)
BSE practices applied	
Inspection	3 (1.4)
Palpation	116 (53.6)
Inspection and palpation	98 (45)
Knowledge about which arm to be used for BSE	
Right hand for left breast and vice versa	33 (15)
The same arm for the same side breast	13 (6)
Any (no protocol)	171 (79)

### Knowledge of the right age to perform BSE and the reasons given to perform BSE

Those performing BSE had different perspectives on the correct age to commence BSE, which should be between 10 and 30 years of age (mean age 18.41 ± 2.8 SD). Of these, 63 recommended BSE at 20 years and 144 responded “I don’t know”. Of those who had performed BSE, 107 respondents reported no specific time/any time they could remember, and 133 reported practicing it on a regular basis (Table 3).

**Table 3:** Distribution of time BSE practiced, and the reasons given to perform or not perform BSE among women in Wolaita Sodo, 2019 (*n* = 626)

Variable/characteristics	Frequency (%)
Appropriate time of BSE	
Few days after menses	97 (45)
Few days before menses	13 (6)
No specific time	107 (49)
Frequency of BSE practices	
Twice per month	48 (22.2)
Once every month	98 (45)
Once every 6 month	2 (0.9)
Once per year	4 (1.9)
Any time I observe a change	65 (30)
Advantage of regular breast self-examination	
Detect any abnormality	72 (33)
Learn how the breast normally looks and feels	56 (26)
Detect breast cancer earlier and promote treatment	89 (41)
Reasons for performing BSE	
Fear of breast cancer	51(23.5)
Early detection of breast cancer	128 (59)
Breast cancer in the family/friends	13 (6)
Previous breast problems	3 (1.4)
Heard from media	22 (10)
Barriers to BSE	
I don’t have enough privacy for BSE practice	14 (6.4)
Pressure of work/I am too busy	30 (13.8)
Doubts about its effectiveness	11 (5)
Absence of symptoms/disease	13 (6)
I am scared of being diagnosed with breast cancer	7 (3.4)
Forgetfulness	10 (4.6)
I know I can never have breast cancer	13 (6)
No obstacle (barriers)	119 (54.8)

### Reasons for not performing BSE

**Fig. 3:**
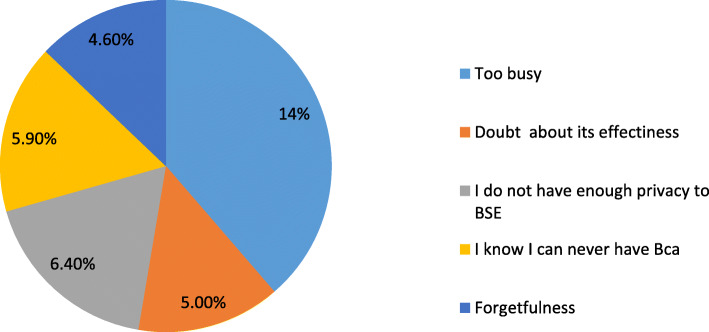
Reasons of not performing Breast self-examination

Of respondents who had ever heard of BSE, 45 believed that they had some kind of barrier to practicing BSE. However, over half of performers (54.8%) claimed that there was no obstacle to performing BSE (Fig. 3).

### Distribution of spousal/parents support to perform BSE and the need of information on BSE

Spousal and other support for BSE was 67.2%. However, 88% of BSE performers were confident in performing self-examination. Almost all study participants (98.6%) knew early detection of breast cancer improved the chances of survival. 91% of respondents wanted more information about BSE. Within the year before this study, among performers of BSE, 149 participants performed it more than six times and 26 participants did it four to six times (Table 4).

**Table 4:** Distribution of spousal/parental support to perform BSE and the need for further information among women in Wolaita Sodo, 2019 (*n* = 626)

Variables/characteristics	Frequency (%)
Support on BSE from spouse/partner	
Yes	146 (67.2)
No	71 (32.8)
Would like information on how to do BSE	
Yes	249 (91)
No	23 (9)
Recognized importance of BSE	
Very important	207 (95.3)
Important	10 (4.7)
Self-confidence to perform BSE	
Yes	191 (88)
No	26 (11)
Where will you go, if you discover a breast lump	
Health facility	168 (77.4)
Traditional healer	49 (22.6)

### Factors associated with BSE

Bivariate binary logistic regression analysis revealed that occupational status, duration of breastfeeding, female education, husband’s education, early detection method for breast cancer, source of information, and knowledge of personal history of having a benign breast lump were candidates for multiple logistic regression analysis at *p* ≤ 0.25.

In the multivariable logistic regression analysis, occupational status of women, duration of breastfeeding, and previously heard of BSE were significantly associated with performing BSE (*p* < 0.05). Women who had mentioned BSE as a method for the early detection of breast problems were 6.36-times (95% CI: 3.72, 10.71) more likely to perform BSE than those reported not knowing any method. Those who had breast fed for 13–24 months were 2.43-times (95% CI: 1.28, 4.59) more likely to examine their breasts than those who mentioned different categories/duration of breast feeding. Employed study participants were 3.13-times more likely (95% CI: 1.14, 8.58) to practice BSE than those who were unemployed. Likewise, students were 3.73-times (95% CI: 1.19, 11.73) more likely to perform BSE than others (Table 5).

**Table 5:** Factors associated with breast self-examination among women in Wolaita Sodo, 2019 (*n* = 626)

Variables	Perform BSE		Odds ratio (95% CI)	
	Yes	No	COR (95% CI)	AOR (95% CI)
Participant’s occupation status				
House wife	96 (15%)	217 (34.4%)	1.00	1.00
Employee	76 (12%)	57 (9%)	2.07 (1.20, 3.59)	3.13 (1.14, 8.58)
Merchant	26 (4%)	49 (7.7%)	6.25 (3.42, 11.41)	6.47 (2.31, 18.12)
Student	19 (3%)	89 (14%)	2.49 (1.25, 4.94)	3.73(1.19, 11.73)
Duration breastfeeding				
Birth-12 months	39 (7.8%)	39 (7.8%)	1.00	1.00
13–24 months	101 (20%)	181 (36.4%)	2.10 (1.18, 0.74)	2.43 (1.28, 4.59)
25–34 months	46 (9.2%)	92 (18.4%)	1.16 (0.75, 0.78)	1.19 (0.74, 1.92)
Early detection method for breast cancer				
BSE	111 (32%)	9 (33%) 364	7.03 (4.14, 11.95)	6.36 (3.72, 10.71)
I don’t know	106 (20%)	(59%)	1.00	1.00
Personal history of having benign breast lump				
Yes	21 (3.5%)	59 (10%)	2.31 (1.20, 4.46)	0.03 (0.08, 1.52)
No	196 (33%)	315 (53%)	1.00	1.00
Women’s educational status				
Primary	82 (28.2%)	209 (71.8%)	1.00	1.00
Secondary	135 (39.9%)	203 (60.1%)	1.70 (1.21, 2.37)	0.81 (0.29, 2.24)
Husband’s educational status				
Primary	51	118	1.00	0.62 (0.26, 1.49)
Secondary	134	175	1.80 (1.21, 2.67)	
Source of information				
Electronics media	151 (25.5%)	218 (36.8)	1.63 (1.14, 2.32)	1.5 9(1.01, 2.59)
Other	66 (11%)	156 (26.3)	1.00	1.00

Adjusted odds ratio (AOR), Significant at *p*- ≤ 0.05

## Discussion

This study showed that, in a random sample of women in an Ethiopian city, 94% of respondents had ever heard or read about breast cancer. This figure is higher than the 83% reported for women in Mekelle town, northern Ethiopia [10] and lower than a study of female Malaysian students at 99.5% [12]. These differences might reflect different educational levels among the study participants and/or differences in the study settings. We also found that 46% of women had previously heard about BSE, lower than in several other studies conducted in other countries: 78.4% in the Malaysian study [13], 67% in Jordanian women [14], 47% in undergraduate students in a teacher training college in Cameroon [15], 80.9% in Chinese immigrants [1], and 72.1% in women in a rural area of western Turkey [16]. However, awareness of BSE in our study was higher than in Benghazi, Libya, which showed that only 41.5% of women had heard of BSE [17]. These differences are likely to represent differences in socioeconomic and demographic characteristics of the study populations.

The relatively low knowledge of our respondents about BSE might prevent them from performing BSE, which might reduce chances of early detection of the disease. 62% of our participants who had received breast cancer information indicated that their main source of information was from the media, with colleagues and friends also mentioned as important sources of information about breast cancer. Amazingly, the proportion of respondents who mentioned health professionals as a main source of breast cancer information was only 13.8%. This is consistent with findings from a similar study conducted in Jordanian females where relatives, friends, and neighbors were found to be the main sources of breast cancer information [14] but different to a study of Iranian women, where health professionals were the main source of information (32.4%) [18].

In the present study, 98% of breast cancer-informed participants knew that early detection of breast cancer improves chances of survival from the disease. This finding is supported by the study of Mekelle town women [10] and higher than a previous in western Ethiopia (74.7%) [19]. Among respondents who reported to have had information on BSE, 79% had at some point performed BSE, lower than in a study of Nigerian Nurses in Lagos General Hospital (89%) [4], and higher than studies of women in northern Ethiopia (37.3%) [20], female Malaysian students (25.5%) [12], female traders in Ibadan, Nigeria (18%) [6], women in a rural area of western Turkey 40.9% [16], and female household heads in northern Ethiopia (53.6%) [10]. Our result was similar to that reported for female health professionals in Welega (77%) [19]. Forty-five percent of our study sample performed BSE on a regular monthly basis, which is higher than in Jordanian women (only 7%) [14], female Malaysian students (31.2%) [12], and female undergraduate students in a teacher training college in Cameroon (25.9%) [15]. This could be due to the different periods when these studies were conducted.

Twenty-nine percent of our participants knew the correct age at which BSE should be started, slightly greater women in Benghazi, Libya (27.7%) [17] but fewer than those in south east Asia, where 44% of study participants recommended practicing BSE at age 20 [5], Nigerian women (60.28% recommended age 20) [21], and women in Kyadondo County, Uganda (40%) [22].

The different breast screening methods recognized by participants in the present study were BSE in 15.4% of women, clinical breast examination in 42.4%, and mammography in 0.3%.

Canadian women knew about BSE (64.3%), clinical breast examination (45.7%), and mammography (32.7%) [20]. A study performed in northern Ethiopia on breast cancer screening methods reported by health extension workers were BSE (14.4%), clinical breast examination (22.3%), and mammography (3%) [23]. These differences may be due to different educational levels and participant groups.

In this study, 53.6% of women conducting BSE had support from their partners, which was higher than another study reporting that 39.8% of women performing BSE were getting support from their spouses/partners [24].

The major barriers to practicing BSE identified in the present study were pressure of work/being too busy, not having enough privacy to perform BSE, thinking that breast cancer wasn’t possible, forgetfulness, and doubt about its effectiveness in 30 (13.8%), 14 (6.4%), 13 (5.9%), 10 (4.6%), and 11(5%) of respondents, respectively. However, over half of women performing BSE (119; 54.8%) could not identify any obstacle to performing BSE. A study conducted in western Ethiopia reported that no current breast problem (12.7%), not feeling comfortable performing BSE (2.7%), scared of being diagnosed with breast problem or cancer, not believing it is beneficial (4%), and not knowing how to do it (7.7%) were barriers to not performing BSE [19].

In another study, the three main reasons for not performing BSE were no breast problem (53.2%), not knowing the BSE technique (30.6%), and not knowing the importance of BSE (21.4%) [23]. In a study of female Debre Birhan University students, the main reasons for not performing BSE were a lack of knowledge on how to conduct BSE and not having any symptoms of breast cancer [25]. A study of female household heads in northern Ethiopia indicated that the major barriers to practicing BSE were an absence of symptoms and lack of knowledge about its importance [10].

Being healthy (44.8%) and a lack of knowledge about BSE (26.9%) were the most significant barriers mentioned for not practicing BSE at Adama Science and Technology University [11].

The current study revealed that women who recognized BSE as an early breast cancer detection method were 6.36-times more likely to practice BSE than women who did not know of any methods to detect breast cancer. This finding is consistent with a study of women in Malaysia, which showed that knowledge that BSE is an early detection method for breast cancer was significantly associated with BSE [13].

In the current study, being employed in an occupation other than being a housewife was significantly associated with performing BSE, with these women 3.12-times (95% CI: 1.14, 8.58) more likely to practice BSE than other groups. These results are in agreement with findings in Nigerian women [26], a study in Benghazi, Libya [17], and a study from southern Ethiopia [24]. This may due to these other occupations exposing these women to a wider selection of media, friends, and colleagues to share ideas and experiences and initiating BSE practice.

Women who had breast fed their child for 13–24 months were 2.43-times more likely to examine their breasts than those who mentioned different durations of breast feeding, which may be due to those who optimally breast feed being more conscious of or educated to perform BSE.

Women who used electronic media as a source of information were 1.59-times more likely to practice BSE than women who used other media types. This may be due to its relative accessibility compared to other source of information.

### Strengths and limitations of the study

The main strength of the study is that previous studies conducted in Ethiopia focused on health professionals, whereas we studied a general urban community. However, this study was limited by being conducted in a single urban community, which may not be representative of the rural community or other urban communities in Ethiopia. Since this was a cross-sectional study, causal conclusions cannot be drawn.

## Conclusion

In general, a low proportion of participants had previously heard about BSE. Only about half of participants performed BSE regularly, and less than a third of respondents correctly recognized the age at which BSE should commence. The use of electronic media as a source of information, occupation, and knowledge about early detection methods for breast cancer were associated with performing BSE. Therefore, we recommend that the Wolaita Sodo administration needs to use electronic media consistently and programmatically (e.g., Wolaita Sodo Fana FM, Wolaita Sodo Wogeta FM, and South TV) to advocate performing BSE. Weekly or monthly mobile phone messages could be sent to encourage performing BSE. Video screens could be used in the city center to demonstrate BSE issues. The advantage of performing BSE over other early screening methods must also be emphasized in public health campaigns.

## Data Availability

The data for this research is available on request. Someone who wants data of this study can contact corresponding author.

## Abbreviations

AOR: adjusted odds ratio
BSE: breast self-examination
CBE: clinical breast examination
CI: confidence interval
OR: odds ratio
